# Complete Genome Sequence of a Multidrug-Resistant Klebsiella pneumoniae Environmental Isolate from Zanzibar, Tanzania, Harboring Novel Insertion Elements and Two *bla*_CTX-M-15_ Genes

**DOI:** 10.1128/mra.00263-22

**Published:** 2022-07-14

**Authors:** Claire Lallement, Karen A. Krogfelt, Ole Skovgaard, Lotte Jelsbak

**Affiliations:** a Department of Science and Environment, Roskilde University, Roskilde, Denmark; Loyola University Chicago

## Abstract

Here, we report the annotated whole-genome sequence of Klebsiella pneumoniae strain KP_3b, isolated in Zanzibar, Tanzania, from plastic litter. The strain is extended-spectrum β-lactamase (ESBL) producing and multidrug resistant, encoding 17 resistance genes, most of which are located on a 230,544-bp plasmid. The isolate contains two copies of the *bla*_CTX-M-15_ gene and novel insertion elements.

## ANNOUNCEMENT

Klebsiella pneumoniae strain KP_3b was isolated from plastic litter collected in Mtopepo (GPS coordinates, 6°08′46.0″S, 39°13′14.5″E), in Zanzibar, Tanzania, by cultivation on selective LB agar plates containing ciprofloxacin (1).

To investigate the context of the numerous drug resistance genes and insertion elements (IS) found in this strain (1), we report here the complete genomic sequence of Klebsiella pneumoniae strain KP_3b, assembled from newly obtained long reads combined with BGISEQ short reads obtained previously (1). Further details of the isolation site, cultivation, and DNA extraction can be found in reference 1. Long reads were obtained using an Oxford Nanopore MinION FLO-MIN106D R9.4.1 flow cell loaded with a library prepared using the SQK-LSK109 ligation kit, minimizing the shearing of DNA. Fast5 files were base called to fastq files using Guppy v5.0.7 with the dna_r9.4.1_450bps_sup model, yielding a total of 1,253,844,477 nucleotides (nt) in 128,252 reads (*N*_50_, 14,277 nt). Reads longer than 20,000 nt were assembled into scaffolds with Flye v2.9 (2) or with iterations of the Minimap, Miniasm (3), and Racon (MMR) assembly (4). Both methods resulted in two contigs of 5,331 kbp and 230 kbp with a mean coverage of 64×. A blastn pairwise alignment of the assembled contigs indicated that they were congruent but deviated at 565 positions. The Flye assembly results indicated that these contigs are circular. The MMR assembly was polished with the BGISEQ short reads (BGI) (5,804,065 paired-end reads; length, 150 nt; coverage, 120×) using SPAdes v3.15, with the –isolate and –trusted-contigs parameters set (5). Finally, we resolved 13 indels, introduced by SPAdes and ranging from 76 bp to 211 bp, by alignment of the SPAdes polished sequence with the trusted MMR assembly. The contigs were manually rotated to encode DnaA (chromosome) and RepB (plasmid) as the first coding DNA sequences (CDS), respectively. The GC content is 57.4% for the chromosome and 51.6% for the plasmid. We annotated the sequences using PGAP v2021-07-01.build5508 (6) with default settings.

As reported previously (1), the isolate contains 16 resistance genes, including one extended-spectrum β-lactamase (ESBL) *bla*_CTX-M-15_ gene. However, in the present assembly, PGAP annotation indicated that the isolate contains a *bla*_CTX-M-15_ gene both on the chromosome and on the plasmid. Both the chromosomal and plasmid copies of *bla*_CTX-M-15_ are associated with IS*Ecp1* (7). Point mutations in the efflux pump regulators *acrR* and *ramR* (8) and the porin genes *ompK36* and *ompK37* (9) were identified using ResFinder analyses (10). Thirty IS elements were annotated on the plasmid. Seven copies of IS*26* (11) as well as two copies of IS*5075* affiliated with antibiotic resistance genes were found on the plasmid. Three new IS candidates were identified (two plasmid borne, one chromosomal). These have been submitted to the ISfinder database (IS*Kpn91*, IS*Kpn92*, IS*Kpn93*) (12). The isolate is multidrug resistant (Table 1).

**TABLE 1:** MIC data for the indicated antimicrobials

Antibiotic(s)	MIC (μg/mL)^*a*^	Phenotype^*b*^
Amoxicillin + clavulanic acid	32	R
Cefepime	16	R
Ceftriaxone	>128	R
Imipenem	<0.125	S
Ciprofloxacin	4	R
Cefuroxime	<0.25	I
Amikacin	4	S
Tigecycline	>16	R
Kanamycin	256	R
Streptomycin	>256	R
Gentamicin	8	R
Piperacillin/tazobactam (6 μg/mL)	64	R

a MICs were measured using broth microdilution according to the EUCAST guidelines.

b Phenotypes (R, resistant; I, intermediate; S, sensitive) are based on clinical breakpoints according to the EUCAST guidelines.

### Data availability.

The complete genome sequence of Klebsiella pneumoniae strain KP_3b has been deposited at GenBank under accession numbers CP086724 (chromosome) and CP086725 (plasmid). The raw BGISEQ reads and the base-called Oxford Nanopore reads have been deposited in the NCBI SRA database under accession numbers SRX7403512 and SRX13090583, respectively, and BioProject accession number PRJNA596383.
